# Drug-induced keratin 9 interaction with Hsp70 in bladder cancer cells

**DOI:** 10.1007/s12192-018-0913-2

**Published:** 2018-05-25

**Authors:** C. Andolino, C. Hess, T. Prince, H. Williams, M. Chernin

**Affiliations:** 1 Biology Department, Bucknell University, 1 Dent Drive, Lewisburg, PA 17837 USA; 2 Department of Urology, Geisinger Clinic, Danville, PA USA

**Keywords:** Keratin 9, Hsp70, Bladder cancer, VER155008

## Abstract

A pull-down experiment (co-immunoprecipitation) was performed on a T24 human bladder cancer cell lysate treated with the Hsp inhibitor VER155008 using an Hsp70 antibody attached to Dynabeads. Keratin 9, a cytoskeleton intermediate filament protein, was identified by LC MS/MS analysis. This novel finding was confirmed by Western blotting, RT-PCR, and immunocytochemistry. Other members of the keratin family of proteins have been shown to be involved in cancer progression, most recently identified to be associated with cell invasion and metastasis. The specific role of keratin 9 expression in these cells is yet to be determined.

## Introduction

Normal cells are able to survive in stressful conditions by increased expression of heat shock protein (Hsp) genes. Overexpression of these genes is also seen in various disease states such as cardiovascular diseases, Alzheimer’s disease, amyotrophic lateral sclerosis, and cancers. In cancers, Hsps facilitate rapid cell proliferation, inhibition of apoptosis, and the promotion of the spread of cancer (Murphy 2013).

Six families of Hsps have been described based on their molecular weight. These include Hsp100, Hsp90, Hsp70, Hsp60, Hsp40, and the small Hsps, including Hsp27. They are referred to as molecular chaperones due to their facilitation in folding newly synthesized or misfolded proteins. Recent data have shown that the correct folding of proteins depend on the sequential cooperation of various Hsps. For example, the role of Hsp in the activation of client proteins such as the glucocorticoid receptor begins with the receptor binding to a complex containing Hsp70 and Hsp40 (Cavanaugh et al. 2015; Wegele et al. 2006; Hernandez et al. 2002; Lindguist and Craig 1988).

Both Hsp90 and Hsp70 are upregulated in various cancers, including bladder cancer. It has been shown that in vitro treatment of bladder cancer cell lines with Hsp90 inhibitors reduce cell growth and upregulate other Hsps, most notably Hsp70. In the bladder cancer cell lines that have been tested, Hsp70 expression is increased five–tenfold by treatment with Hsp90 inhibitors (Cavanaugh et al. 2015).

In order to identify Hsp70 client proteins in bladder cancer cells, a co-immunoprecipitation experiment was performed with cell lysates, and keratin 9 was identified from a lysate treated with VER155008, an Hsp70 inhibitor. Keratin 9 is a member of a subgroup of type I intermediate filaments which is traditionally only expressed in the palms of the hands and soles of the feet (Moll et al. 2008). The identification of keratin 9 as a client protein of Hsp70 raises some fundamental questions. Why is this protein expressed in human bladder cancer cells treated with VER155008 and what role, if any, does keratin 9 plays in the progression of bladder cancer? Other keratins have been identified that play significant roles in cancer, specifically cell invasion and metastasis (Chivu-Economescu et al. 2017; Obermajer et al. 2009). Here, we present evidence of the novel result that keratin 9 is an Hsp70 client protein and is expressed in human bladder cancer cells.

## Materials and methods

### Cell culture and lysate preparation

Bladder cancer cell line T24 was purchased from ATCC. Cells were grown in EMEM plus L-glutamine (ATCC) supplemented with 10% fetal bovine serum (Atlanta Biologicals), 10 units of penicillin, 10 μg streptomycin and 0.025 μg/mL amphotericin B (Sigma). Control (DMSO) and Hsp70/Hsp90 inhibitor-treated cell lysates were prepared for the co-immunoprecipitation and immunoblotting by incubating cells in 1.0 mL of CelLytic M (Sigma) for 10 min at room temperature.

### Antibodies and reagents

The Hsp70 and the keratin 9 antibodies used for each experiment were obtained from StressMarq Biosciences and Santa Cruz Biotechnologies, respectively. For immunocytochemistry, Alexa Fluor 488 Dye (Molecular Probes) was used to allow for visualization via immunofluorescent confocal microscopy.

### Co-immunoprecipitation

Co-immunoprecipitation of Hsp70 from T24 bladder cancer cell lysates was carried out utilizing the Immunoprecipitation Kit Dynabeads Protein G (Novex Life Technologies). The protocol was followed according to the manufacturer’s instructions, beginning with the addition of 10 μL of Hsp70 antibody (StressMarq) or 10 μL of mouse IgG (Santa Cruz Biotechnology) to create the Co-IP bead complex. T24 human bladder cancer cells (80% confluency) were treated with DMSO or VER155008 (Tocris) for 48 h and cell lysates prepared. Each lysate (500 μL) was added to the Hsp70 antibody or mouse IgG-Dynabead complex and incubated for 10 min. The bound proteins were eluted and the samples were electrophoresed. The gel was silver stained following the SilverQuest Novex protocol and a single band from the +VER1555008 lysate was excised and sent to Bio-Synthesis, Inc. to obtain a LC-MS/MS Protein Identification Report.

### RT-PCR

Total RNA was isolated from T24 human bladder cancer cells using TRIzol according to the manufacturer’s instructions (Thermofisher). Contaminating DNA was removed with DNase I (Promega) and RNA quality was analyzed in a 1% agarose gel. Reverse transcription was performed using 1 μg of RNA in a OneStep RT-PCR Kit (QIAGEN). Keratin 9 gene sequences were amplified using specific primers (Fig. 3) and PCR products were analyzed in a 2% agarose gel.

### Immunoblotting

Cell lysates treated with DMSO (vehicle), VER155008 (VER), STA9090 (STA) (MedChem Express) and MAL3-101 (MAL) were resolved on a Bolt 4–12% Bis-Tris Plus gel (Invitrogen), and transferred to a nitrocellulose membrane via iBlot 2 Dry Blotting System (Life Technologies). The blocked membrane was incubated 12 h with a 1:500 dilution of mouse monoclonal keratin 9 antibody (Santa Cruz Biotechnologies). Blots were then washed and incubated with anti-mouse IgG, HRP-linked secondary antibody (cell signaling) according to the manufacturer’s instructions. The membrane was simultaneously probed with a mouse monoclonal anti-GAPDH antibody (Santa Cruz Biotechnology) as an internal loading control. The blot was visualized by C-Digit Blot Scanner (LI-COR) after enhancing it with SuperSignal West Pico Chemiluminescent Substrate (ThermoFisher Scientific).

### Immunofluorescence

T24 human bladder cancer cells were grown on microscope coverslips in a cell culture dish for 48 h. The cells were fixed in 100% methanol at − 20 °C for 10 min and processed for immunocytochemistry. Cells were permeabilized in 0.5% triton X-100/PBS for 15 min at room temperature then incubated overnight at 8 °C with a 1:500 dilution of a mouse monoclonal anti-keratin 9 antibody (Santa Cruz Biotechnology) or a 1:1000 dilution of a mouse monoclonal anti-HSP70 antibody (StressMarq). After successive washings in PBS, the cells were incubated with a 1:400 dilution of an anti-mouse Alexa Flour 488 conjugate (Molecular Probes) for 2 h at room temperature. The coverslips were attached to slides with Vectashield Hardset Mounting Media containing DAPI and inspected in a Leica SP5 confocal microscope. Negative controls were incubated without primary antibody.

## Results

### Immunoprecipitation and LC-MS/MS analysis

**Fig. 1:**
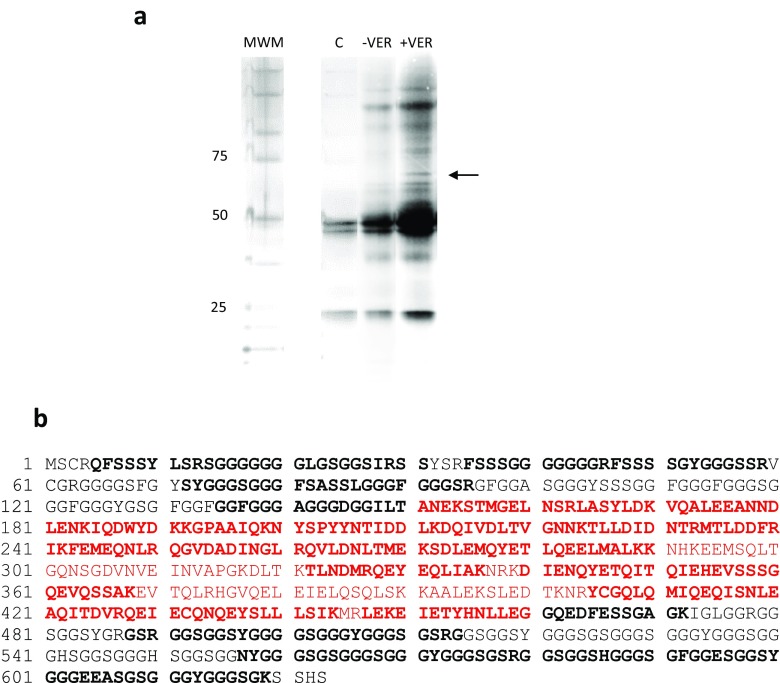
Hsp70 and IgG antibodies were linked to Dynabeads, and T24 bladder cancer cell lysates were immunoprecipitated. SDS-PAGE, followed by silver staining, was performed and the band at the arrow was sliced from the gel and analyzed by LC-MS/MS. The unknown band was determined to be keratin 9. **a** Hsp70-Dynabead-linked Immunoprecipitation of T24 lysates in the absence of VER155008 (VER-) and presence of VER155008 (VER+). Lane C is an IgG-Dynabead-linked immunoprecipitate of a DMSO-treated T24 lysate (IgG control). Molecular weight markers (MWM) of 25, 50, and 75 represent molecular weights in kilodaltons. **b** bold black and red letters represent keratin 9 amino acid fragments identified from the excised band by LC-MS/MS. Red letters identify the amino acids found in the filament super family

To identify proteins that interact with Hsp70 in bladder cancer cells, we performed a pull-down assay using a lysate treated with the Hsp70 inhibitor VER. The VER-treated lysate was chosen at random as we had several lysates treated with Hsp70 and Hsp90 inhibitors. The results of this experiment can be seen in Fig. 1a. It is apparent that several proteins were pulled down from lysates in the absence of VER (-VER) and presence of VER (+VER). We chose the band located at the arrow for LC-MS/MS as it was present only in the +VER lysate. The dark doublet bands (50 kD) in lane C (control pull-down with mouse IgG antibody), in addition to the band at 25 kD, are consistent with mouse IgG subunits under denaturing conditions. The mouse IgG 50 and 25 kD bands are also present in -VER and +VER lysates. The LC-MS/MS results yielded 22 amino acid fragments from the excised band. Alignment of the fragments and a Uniprot Swisspro Trembl database search (performed by Bio-Synthesis, Inc.) identified keratin 9 as a likely match. Our analysis using BLASTP and matching the amino acid sequence in the fragments with the known amino acid sequence of keratin 9 is shown in Fig. 1b. The bold black and red letters represent amino acid fragments identified from the excised band by LC-MS/MS. The bold amino acids represent 69% of the overall sequence. Keratins share a central domain known as the filament super family (red) flanked by unique head and tail domains (Omary et al. 2009). Comparison of amino acid sequences between keratin 9 and keratins 1, 10, 4, and 14 show 16% identity only in the filament superfamily.

### Western blot analysis

**Fig. 2:**
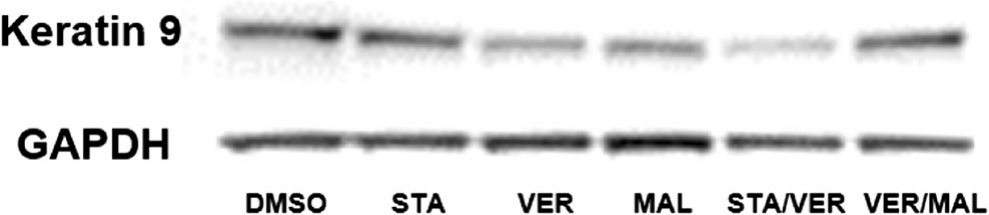
Western blot analysis of human bladder cancer cell lysates (T24) treated with DMSO, the Hsp90 inhibitor STA9090, the Hsp70 inhibitors VER155008, and MAL3-101 or combinations of the various inhibitors. The blot was probed with a keratin 9 mouse monoclonal antibody

Since the VER-treated T24 bladder cancer cell lysate contained what we suspected to be keratin 9 as a client protein of Hsp70, we wanted to confirm keratin 9 expression in other Hsp inhibitor-treated T24 bladder cancer cells by Western blot analysis. The blot contained lysates treated with DMSO (vehicle control), STA (Hsp90 inhibitor), VER (Hsp70 inhibitor), and MAL (Hsp70 inhibitor) and in combination (Fig. 2). The expression of keratin 9 closely mimicked results which showed that muscle invasive bladder cancer cell lines were more sensitive to dual Hsp70 inhibition as compared to monotherapy with either Hsp70 or Hsp90 monotherapy agents with the exception of VER/MAL (Cavanaugh et al. 2015).

### RT-PCR

**Fig. 3:**
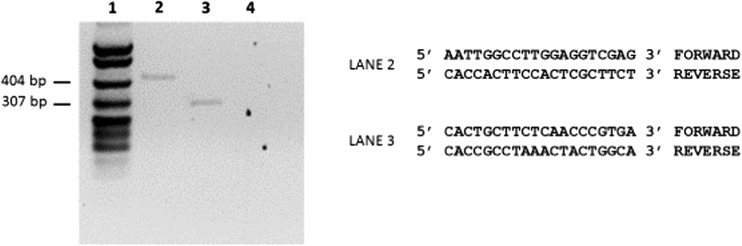
RT-PCR of RNA isolated from T24 human bladder cancer cells. Lane 1, *Msp*I digest of pBR322 used as a molecular weight marker. Lane 2, amplified PCR product (416 bp) using forward and reverse primers shown on the right (lane 2). Lane 3, amplified PCR product (300 bp) using forward and reverse primers shown on the right (LANE 3). Lane 4, PCR reaction in the absence of RNA (negative control)

Based on the known mRNA sequence of keratin 9, forward and reverse primers were designed complementary to nucleotides between positions 32 and 51 (forward primer) and positions 312 and 331 (reverse primer). The resulting amplification product (300 bp) was located within the unique head region of keratin 9. Further confirmation was obtained by amplifying a region within the unique tail region of keratin 9. The forward primer was complementary to positions 1482 and 1501 and the reverse primer was complimentary to positions 1878 and 1897 which yielded a PCR fragment of 416 bp. These results are depicted in Fig. 3.

### Immunocytochemistry

**Fig. 4:**
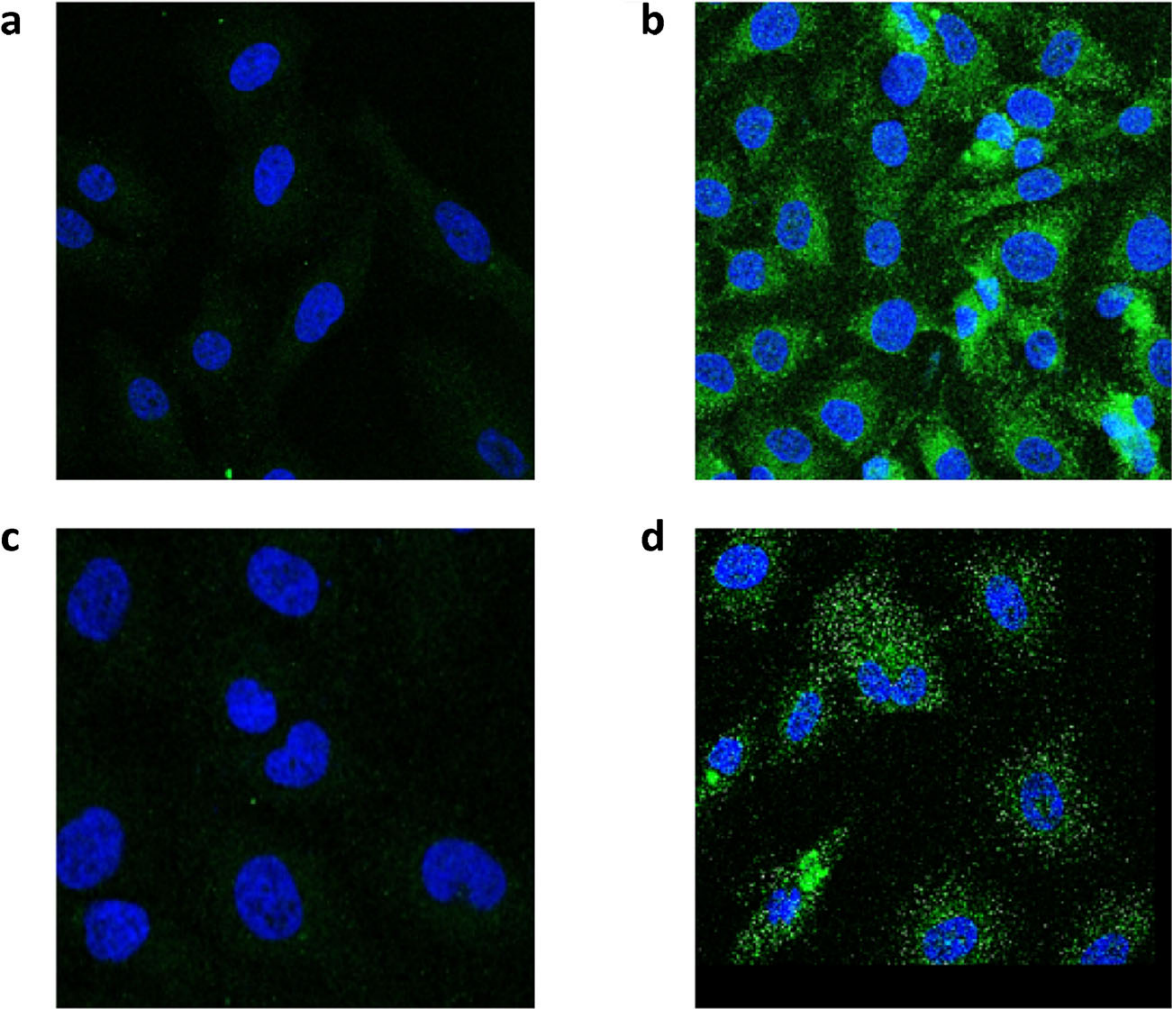
Immunostaining using keratin 9 and Hsp70 mouse monoclonal antibodies and visualized by confocal microscopy. T24 human bladder cancer cells were processed as described in “Material and methods.” The negative controls without primary antibodies (**a**, keratin 9) and (**c**, Hsp70) show slight background staining resulting from autofluorescence of the secondary antibody. The nuclei were stained with DAPI. Perinuclear distribution of keratin 9 (**b**) and punctate distribution of Hsp70 (**d**)

Biochemical (LC-MS/MS) and molecular analysis (Western blot and RT PCR) provided credible evidence that keratin 9 was the protein isolated from the band in the co-immunoprecipitation experiment. To confirm that keratin 9 was expressed in the T24 bladder cancer cells, immunostaining was conducted to assess the subcellular distribution of keratin 9 and Hsp70, using a keratin 9 monoclonal antibody and the same Hsp70 monoclonal antibody used in the co-immunoprecipitation experiment (Fig. 4). Cells were processed as described in “Material and methods.” The negative controls with no primary antibody (Fig. 4a, c) show slight background staining resulting from autofluorescence of the secondary antibody. The nuclei were stained with DAPI present in the mounting media. Figure 4b shows enriched perinuclear distribution of keratin 9, a staining pattern that is consistent with other members of the keratin family (Lee et al. 2012). Figure 4d shows more diffuse, punctate, and cytoplasmic staining of Hsp70 typical of heat shock proteins.

## Discussion

The experiments presented above clearly demonstrate that keratin 9 binds to Hsp70 in the presence of VER, a potent inhibitor of the Hsp70 family of chaperones which have been shown to contribute to cancer cell survival via anti-apoptotic functions (Massey et al. 2010). The literature on keratin 9 is limited and data indicate that expression of keratin 9 is primarily in the palmoplantar epidermis (Moll et al. 2008). A recent paper suggests that keratin 9 may serve as a biomarker for Alzheimer disease, be implicated in polycystic ovary syndrome (Richens et al. 2016; Kim et al. 2013) and may serve as a marker of metastatic hepatocellular carcinoma (Fu et al. 2009). Interestingly, several simple epithelial keratins (SEKs) can also serve as tumorigenic markers as well as stress proteins (Omary et al. 2009; Toivola et al. 2010). The question as to why keratin 9 is an Hsp70 associating protein in the presence of VER and is expressed in human bladder cancer cells needs to be addressed. VER is an adenosine-derived competitive inhibitor of Hsp70. A plausible explanation is that VER binding to Hsp70 could alter the conformation to allow for interaction with keratin 9. It should be noted that the Western blot results presented in Fig. 2 closely conform to Western blot expression of Hsp70 treated with STA, VER, MAL, and in combination except the combined treatment of VER and MAL (Cavanaugh et al. 2015). It is likely that with the inhibition of Hsp70, keratin 9 that is not folded or folded incorrectly is degraded and not available for detection accounting for the decrease in expression in the presence of the Hsp70 inhibitors. This interpretation is consistent with results from Leu et al. (2011) which showed decreased expression of EGFR, AKT, mTOR, and integrin β1 in cancer cells treated with the Hsp70 inhibitor PES.

It has been implicated that Hsp70 can play a role in tumorigenicity by affecting signaling pathways involved in cancer progression (Murphy 2013). Overexpression of Hsp70 has been shown to be a marker in hepatocellular and prostate cancer (Chuma et al. 2003; Abe et al. 2004), metastasis (Hwang et al. 2003; Lazaris et al. 1997), and relevant to our findings, bladder cancer (Syrigos et al. 2003). Mashukova et al. (2009) have shown, using co-immunoprecipitation experiments, that keratin 8 forms a ternary complex with Hsp70 and protein kinase C. This finding confirms that Hsp70 is capable of interacting with intermediate filaments and indicates a potential role of keratins in signaling cascades.

Keratins 5 and 14 have been shown to play a role in proliferation, differentiation, and cancer progression (Alam et al. 2011). Like keratin 9, keratin 17 is not expressed in normal mature epithelia; however, it is expressed in ectodermal embryonic stem cells and in carcinomas (Chivu-Economescu et al. 2017). Additionally, transfection of keratin 17 negative cells with wildtype keratin 17 increased Akt and mTOR activity as well as protein synthesis and hypertrophy (Kim et al. 2006). Interestingly, the down regulation of keratin expression has been shown to contribute to the epithelial-mesenchymal transition, a hallmark of invasive behavior (Seltmann et al. 2013). This study specifically looked at the keratin 5/keratin 14 pair and concluded that keratins help maintain the epithelial phenotype.

It is interesting to note that the Western blot (Fig. 2), RT PCR (Fig. 3), and immunocytochemistry (Fig. 4) results indicate that keratin 9 is expressed in these cells; however, the question as to why keratin 9 is expressed still remains unanswered. Whatever the role(s) of keratin expression in neoplastic cells, the novel expression of keratin 9 in human bladder cancer cells adds yet another player to the complex interactions of intermediate filaments and their potential significance in the tumorigenic process.
